# Clinicopathological Features of Young Versus Older Patients With Breast Cancer at a Single Pakistani Institution and a Comparison With a National US Database

**DOI:** 10.1200/JGO.18.00208

**Published:** 2019-03-12

**Authors:** Sana Zeeshan, Basim Ali, Khabir Ahmad, Anees B. Chagpar, Abida K. Sattar

**Affiliations:** ^1^ The Aga Khan University, Karachi, Pakistan; ^2^ Yale University School of Medicine, New Haven, CT; ^3^ Yale Comprehensive Cancer Center, New Haven, CT

## Abstract

**PURPOSE:**

The age at which women present with breast cancer varies widely among nations, and breast cancer may behave differently in younger women. Differences in clinicopathological characteristics based on age have not been well characterized in Pakistani patients with breast cancer.

**METHODS:**

We conducted a retrospective review of patients with symptoms of breast cancer presenting to Aga Khan University Hospital (AKUH), a large tertiary care center in Pakistan, between 2001 and 2010; we compared young (≤ 40 years) versus older (> 40 years) patients in terms of their clinicopathological characteristics. We also compared this Pakistani cohort with the US population using data from the National Cancer Database (NCDB).

**RESULTS:**

A total of 1,334 patients with breast cancer presented to our center over the 10-year review period. The median age at diagnosis was 50 years, compared with 60 years for patients in the NCDB. In the AKUH cohort, younger patients were significantly more likely than their older counterparts to present with metastatic disease (13.1% *v* 10.8%; *P* < .01). They also were more likely to present with higher-grade tumors (grade 3: 40.1% *v* 28.3%; *P* < .001), have triple hormone receptor–negative phenotype (25.4% *v* 14.1%, *P* < .001), and have positive axillary lymph node involvement (70.9% *v* 57.5%; *P* < .001) compared with older women. Younger and older patients in the AKUH cohort tended to present with higher-stage disease (*P* < .001) and were more likely to have triple hormone receptor–negative disease (*P* < .001), compared with all patients in the NCDB and with those of Indo-Pakistani origin.

**CONCLUSION:**

Young Pakistani women, similar to their Western counterparts, present with more advanced disease and more aggressive tumor biology than their older counterparts.

## INTRODUCTION

Breast cancer is the most common malignancy affecting women worldwide and one of the leading causes of cancer-related deaths.^1^ In the United States, only 5% to 7% of all breast cancers are diagnosed in patients younger than 40 years,^2-4^ whereas in the developing countries of Asia and Africa, this figure is reported to be much higher.^5-7^ In addition, patients with breast cancer in low- to middle-income countries (LMICs) tend to present with advanced-stage disease than those in countries with higher income levels. The etiology of these discrepancies between high-income countries and LMICs in terms of age and stage at presentation is not well understood; hypotheses include racial and genetic differences, varying environmental exposures, diversity in access to screening and treatment, and differences in population distribution, with the LMICs having a younger population secondary to lower median life expectancy and higher birth rate.^5-7^ Patients with breast cancer who receive a cancer diagnosis at a young age are more likely to present at an advanced-stage and with more aggressive disease than their older counterparts, according to many studies conducted in Western populations and in other LMICs.^8-10^ No such comparison has been reported from Pakistan, to our knowledge.

In Pakistan, breast cancer is the most common malignancy affecting women, accounting for approximately 40% of all malignant tumors in the female population.^11,12^ The age distribution of these malignancies and the clinicopathologic features of younger patients compared with those of older patients in this population have not been well studied. We sought to determine whether the clinicopathologic features differentiating younger and older patients seen in Western countries would be found in Pakistan and whether this could account for the poorer outcomes in patients with breast cancer in LMICs.

**CONTEXT**

**Key Objective**
  To determine the clinicopathological features of breast cancer in young versus older Pakistani women reporting to a large tertiary care center, and then to compare trends in clinicopathological features of breast cancer in Pakistani women with those of women with breast cancer in the United States and further with those of Indo-Pakistani origin in the United States.
**Knowledge Generated**
  Of Pakistani women with breast cancer, more than 25% present before the age of 40 years. Furthermore, young Pakistani women tend to present with more aggressive and advanced disease compared with young women in the United States and older women in Pakistan.
**Relevance**
  Our findings warrant greater awareness of cancer risk in young Pakistani women and the consideration of screening for breast cancer at a younger age in developing countries that have a greater proportion of younger women.

## METHODS

A retrospective record review was conducted of all Pakistani patients with breast cancer presenting consecutively to the Aga Khan University Hospital (AKUH) in Karachi, Pakistan, between January 1, 2001, and December 31, 2010. Male patients, patients with bilateral breast cancer or multiple malignancies at presentation, and patients who were not of Pakistani descent were excluded from the study. Patients were divided into two groups on the basis of their age at presentation: a cohort of patients younger than 40 years and a cohort of patients older than 40 years. Groups were compared for clinicopathologic features. Staging was performed using the American Joint Committee on Cancer Cancer Staging Manual, Seventh Edition, and receptor status was based on standard immunohistochemical staining.

We also compared this Pakistani cohort with the US population, using 2013 data from the National Cancer Database (NCDB). The NCDB is a joint project of the American College of Surgeons and the American Cancer Society, which collects data from hospital registry data at more than 1,500 Commission on Cancer–accredited facilities in the United States. These data represent more than 70% of newly diagnosed cancer cases in the United States. Data from the 2013 NCDB for patients with breast cancer were used for this comparison. We also analyzed differences between the AKUH cohort and patients of Indo-Pakistani origin in the NCDB. This study was reviewed and approved by the ethics review committee of the Aga Khan University. Nonparametric statistical analyses using Pearson’s χ^2^ test were performed using SPSS, version 24.0 (SPSS, Chicago, IL).

## RESULTS

**FIG 1:**
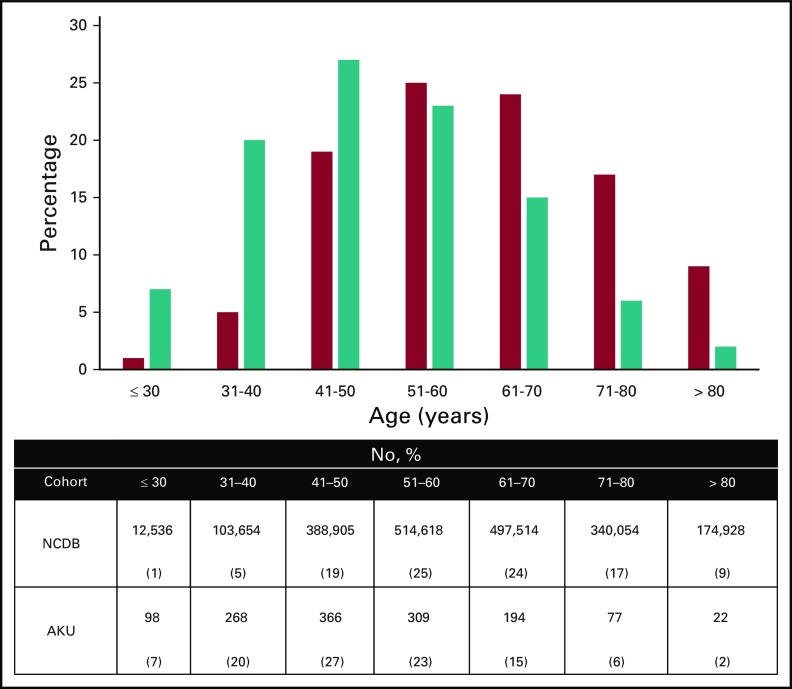
Age distribution of Pakistani versus US population. Teal bars show distribution from Aga Khan University (AKU); red bars show distribution from National Cancer Database (NCDB).

A total of 1,334 patients with breast cancer presented to our center over the 10-year review period. The median age at diagnosis was 50 years (range, 20 to 92 years); 366 patients (27.4%) were younger than 40 years at presentation. The median age at diagnosis in the NCDB was 60 years (range, 18 to 90 years). The two populations were significantly different in distribution (*P* < .001), with the Pakistani population tending to present at younger age (Fig 1).

Clinicopathologic characteristics of the younger and older AKUH cohorts are listed in Table 1. Younger patients tended to present at a higher-stage disease, with lymph node–positive, higher-grade cancers that were less likely to be hormone receptor–positive and more likely to be triple hormone receptor–negative. Analysis of the NCDB data revealed similar correlations between younger age (≤ 40 years) and clinical stage at presentation, lymph node status, grade, estrogen receptor–positivity, and likelihood of having triple hormone receptor–negative disease (*P* < .001 for all comparisons).

**TABLE 1:**
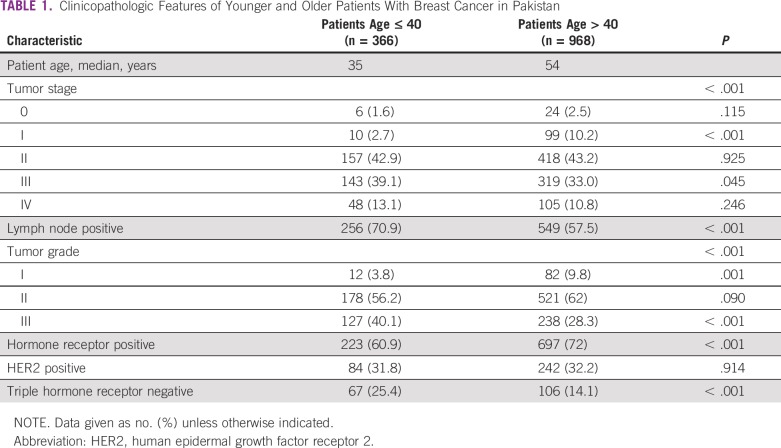
Clinicopathologic Features of Younger and Older Patients With Breast Cancer in Pakistan

Compared with patients in the NCDB, younger (Table 2) and older (Table 3) patients in the AKUH cohort tended to present at higher-stage disease (*P* < .001) and were more likely to have triple-hormone receptor–negative disease (*P* < .001). Patients in both Pakistani cohorts were less likely to present with higher-grade disease than those in the NCDB (*P* < .001). Compared with US patients of Indo-Pakistani origin in the NCDB, patients in the AKUH cohort were significantly more likely to present with advanced-stage and triple negative disease, and were less likely to present with high-grade tumors in both the younger (Table 2) as well as older (Table 3) cohorts.

**TABLE 2:**
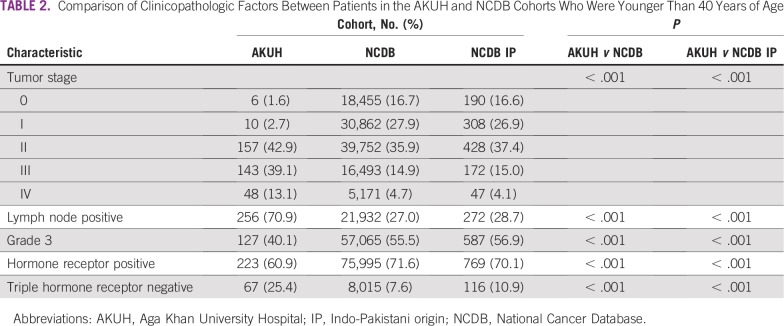
Comparison of Clinicopathologic Factors Between Patients in the AKUH and NCDB Cohorts Who Were Younger Than 40 Years of Age

**TABLE 3:**
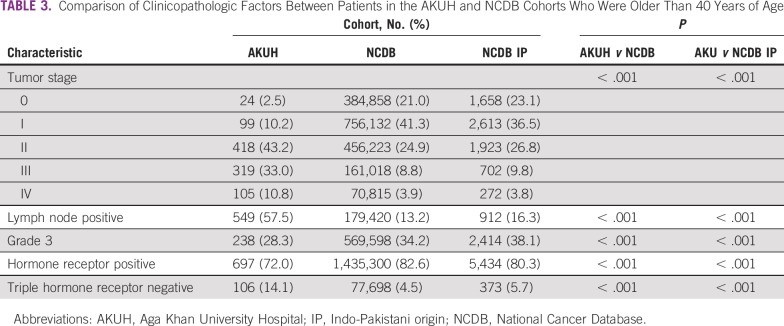
Comparison of Clinicopathologic Factors Between Patients in the AKUH and NCDB Cohorts Who Were Older Than 40 Years of Age

When patients of Indo-Pakistani origin were compared with other races in the US population, using the NCDB data, differences were more evident in the older (> 40 years) cohort. Older patients of Indo-Pakistani origin were more likely to present with higher-grade, triple hormone receptor–negative, node-positive tumors than were older patients of other races in the United States (Table 4). In younger patients, however, breast cancer in patients of Indo-Pakistani origin was of the same stage as other patients, but the former were more likely to present with triple hormone receptor–negative disease (Table 4). There was also a trend for these patients to present with higher-grade tumors.

**TABLE 4:**
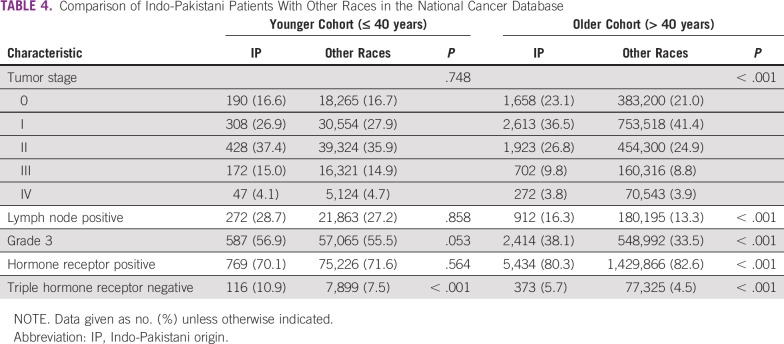
Comparison of Indo-Pakistani Patients With Other Races in the National Cancer Database

## DISCUSSION

The age at which women present with breast cancer varies between countries, but breast cancer generally is thought to be more aggressive in younger women. The median age of breast cancer presentation is much younger in many Asian countries.^5-7^ We found that 27.4% of our institutional cohort was younger than 40 years, which is comparable to findings of other studies from Pakistan,^13,14^ and is a higher rate than the 5% to 21% reported in many Western, African, and Asian studies.^5,6,15^ Indeed, the median age at diagnosis in the NCDB population was a decade older than in the Pakistani cohort, and only 6% of patients in the NCDB received their breast cancer diagnosis at age younger than 40 years. Two reasons may explain these differences. First, in Western countries like the United States, older women (> 40 years of age) are routinely screened with mammography, thereby increasing the detection of breast cancer in older women. Second, the life expectancy of women in Pakistan is significantly lower than that of women in the United States (67.4 *v* 81.0 years, respectively) and the birth rate is significantly higher (3.5 *v* 1.8 births per woman, respectively), resulting in a younger female population in the country. This alone could explain some of the differences in age distribution of breast cancer across the two populations. Differences in genetic and environmental exposures between the two populations could also contribute to these differences; however, data in this realm are lacking.

We found that younger patients in Pakistan were more likely to present at an advanced stage of breast cancer, with more lymph node–positive disease, compared with older women. These findings echo those of others who have studied this concept in other countries. For example, the study by Kroman et al^16^ of 10,356 women from Denmark found that patients with breast cancer who were younger than 35 years had a higher rate of lymph node positivity compared with older women in the study (51% *v* 46%; *P* = .02). Similar results were published by Gajdos et al^17^ from the United States, where women younger than 36 years had larger tumor size with more lymph node involvement; these patients were likely to present with stage II or III disease than were their older counterparts (60% *v* 43%; *P* < .001).

Although some have argued that younger patients may present with more advanced disease because of a lack of awareness and screening, others have speculated this may be because younger patients tend to present with biologically worse disease.^18^ Indeed, younger patients in our institutional cohort, who were not screened, also tended to present with higher-grade tumors that were more likely to be triple hormone receptor–negative. Our study provides a comparison between the Pakistani and US populations, and may provide some insight into this dichotomy. In Pakistan, widespread screening is not available, even for older patients. This has not changed over the 12 years during which our institutional cohort was studied, whereas in the United States, screening is widely available for patients older than 40 years. Patients older than 40 years in the Pakistani cohort presented at a later stage of disease than did patients in the NCDB, suggesting that screening may have an impact. However, this was also the case in the younger cohort, in which routine screening would not play a role, even in the American population. Furthermore, that triple-negative breast cancer is three-fold more common in patients in Pakistan than in the United States, regardless of age cohort, suggests some fundamental differences in the biology of the disease between the two cohorts. Whether this is related to genetic or other factors is unknown.

Our findings when comparing patients of Indo-Pakistani origin with other races in the NCDB would support this hypothesis. In the older cohort, for whom screening is largely available, those of Indo-Pakistani origin tended to present with later-stage disease, with more high-grade, triple-negative tumors. Whether this is due to a reluctance to undergo screening, worse biology, or both is unclear. However, in the younger American cohort, for whom screening is not widely available, patients of Indo-Pakistani origin present at the same stage of disease as their non–Indo-Pakistani American counterparts. However, the incidence of triple hormone receptor–negative disease is greater in the former group. The etiology of this finding, which seems to be ubiquitous across countries and age groups, remains to be elucidated.

Interestingly, the AKUH cohort tended to have fewer high-grade breast cancers compared with the NCDB cohort. However, this may not necessarily reflect more favorable biology in the Pakistani cohort; rather, it may reflect the subjectivity with which grading of breast cancer is done. Pakistani patients were also less likely to have low-grade tumors, compared with their American counterparts (younger cohort, 3.8% *v* 9.5%, *P* < .001; older cohort, 9.8% *v* 22.4%, *P* < .001). Overall, 60% of the Pakistani cases were classified as intermediate grade, whereas the proportion of patients in this category in the US population was 43% (*P* < .001).

To our knowledge, this is the only study performed of young Pakistani women to date in which tumor stage and biology were compared with a control population of older women. Our study should be viewed in light of some limitations. This was a single-center, retrospective study and Pakistani patients at AKUH may not reflect women from all parts of the country. Still, our data have face validity. As in many parts of the developing world, there seems to be a disproportionate number of younger patients with breast cancer in Pakistan compared with more industrialized countries. As noted across populations, younger patients tend to present with higher-stage, lymph node–positive disease and are more likely to have triple hormone receptor–negative disease. However, we found that patients with breast cancer in Pakistan tended to present with higher-stage, lymph node–positive, endocrine-resistant, triple-negative tumors compared with their US counterparts regardless of age at presentation. Our comparison of US patients of Indo-Pakistani origin and their other US counterparts further strengthens the hypothesis that differences in presentation of breast cancer across populations are multifactorial in etiology and not solely due to differences in screening alone. Additional effort to evaluate potential causes of worse biology is required.
